# Rapid resolution of catatonia secondary to post traumatic stress disorder with secondary psychotic features through scheduled zolpidem tartrate

**DOI:** 10.1186/s12888-023-04769-x

**Published:** 2023-04-17

**Authors:** Nicholas Bonomo, Haojiang Huang, Ben Schoenbachler

**Affiliations:** 1 Resident Physician, Department of Psychiatry, University of Louisville, Louisville, KY USA; 2 Director of Norton Hospital Inpatient Unit, Department of Psychiatry, University of Louisville, Louisville, KY USA; 3 Director of Memory Disorders Program, Department of Psychiatry, University of Louisville, Louisville, KY USA

**Keywords:** Catatonia, Post traumatic stress disorder, Post traumatic stress disorder with secondary psychosis, Benzodiazepines, Zolpidem

## Abstract

Catatonia is a complication of numerous psychiatric and medical conditions. The first-line treatment is typically management of the underlying primary condition as well as scheduled benzodiazepines or electroconvulsive therapy. Electroconvulsive therapy and benzodiazepines are not always tolerated or available when treating patients with catatonia. For this reason, other treatment regimens have been trialed in recent years, including the GABA-modulatory Z drugs such as zolpidem. Some alternative treatment modalities have shown great promise. However, which populaces these are most beneficial for is still unclear. In this article, we examine a case report of a woman who suffered from post-traumatic stress disorder with secondary psychotic features who experienced recurrent akinetic catatonia that was refractory to benzodiazepine therapy. She responded rapidly to scheduled zolpidem with minimal side effects. It is our author’s belief that when managing catatonia in patients with post traumatic stress disorder with secondary psychosis, Z drugs may be preferable to benzodiazepines.

## Background

Post-traumatic stress disorder (PTSD) was added to the third edition of the Diagnostic and Statistical Manual of Mental Disorders in 1980 [1]. Despite being a common condition with a global lifetime prevalence of 3.9%, even in the most severe of cases half of individuals never receive any form of treatment [2]. Part of this is due to misdiagnosis, as PTSD can mimic several other conditions such as bipolar disorder which both share sleep disturbances and irritability as common symptoms [3]. In addition, it has recently been proposed that PTSD patients can develop positive symptoms of psychosis, a disorder termed PTSD with secondary psychosis (PTSD-SP) [4].

To meet the criteria for a diagnosis of PTSD-SP the psychotic and mood symptoms must arise after the initiating trauma with no psychiatric illness prior [4]. The positive psychotic symptoms of PTSD-SP can include hallucinations and delusions which may be trauma or non-trauma related [4]. These hallucinations must be distinct from intrusive thoughts or flashbacks [4]. These positive symptoms may resemble primary psychotic disorders, however hallucinations and delusions in PTSD-SP tend to be more paranoid and persecutory in nature rather than bizarre [4]. Avoidance, emotional numbing, affective constriction, detachment and derealization are also common in PTSD in general and may be misinterpreted as negative psychotic symptoms [4]. In severe cases, PTSD may even present with catatonia [5].

Catatonia is a complication of numerous conditions [6]. The most common psychiatric cause is bipolar disorder followed by autism, schizophrenia, and mixed psychiatric illness, with MDD moderating lower catatonia prevalence [6]. It is also seen in numerous medical conditions, such as encephalitis and seizure disorders [6]. Catatonia presents in a variety of manners and is characterized by changes in psychomotor activity [7]. In akinetic catatonia patients are aware of their surroundings but they will stare and are nonresponsive [7]. In excitative catatonia patients have impulsive, often purposeless movements and are prone to agitation and combativeness [7]. In some cases, a syndrome develops characterized by high fevers and autonomic instability termed malignant catatonia which rapidly leads to death if left untreated [7]. Irrespective of manifestation catatonia requires urgent medical intervention, most often through administration of benzodiazepines [7]. In resistant cases electroconvulsive therapy (ECT) is often considered [7]. In most instances these treatments are sufficient, however there are still cases where benzodiazepines and ECT fail. In these situations, other treatments have been investigated including mood stabilizers such as carbamazepine, glutaminergic antagonists such as amantadine, and GABA modulatory z drugs such as zolpidem and zaleplon [8]. Even with promise having been shown for these medications, it is still unclear in which scenarios alternative treatments are preferable. Benzodiazepines are still the mainstay of first line management in most situations.

In the context of PTSD benzodiazepine usage is problematic. PTSD is strongly associated with dissociative symptoms such as derealization [4, 9]. Unfortunately, derealization is a side effect of benzodiazepines [10, 11]. Treating catatonia in PTSD patients may improve catatonia but often worsens dissociative symptomatology, and treatment outcomes are significantly worse for PTSD patients treated with benzodiazepine therapy [12]. For these reasons it may be preferable to avoid benzodiazepines if managing catatonia in PTSD patients.

In this case report we describe the case of a middle-aged female that developed a recurrent psychotic disorder with catatonia as a consequence of her PTSD. This is to say she had PTSD-SP w/ catatonic features. During her most recent hospitalization she presented in a psychotic state with akinetic catatonia. She failed benzodiazepine therapy and was not a candidate for ECT. She responded rapidly when treated with scheduled zolpidem. She was discharged with a short tapering regimen and has maintained over half a year free from recurrent symptoms. It is the authors belief that this case suggests that z drugs such as zolpidem may be superior to benzodiazepines when treating catatonia in PTSD-SP patients.

## Case presentation

Jane was a middle-aged female with a history of catatonia, hypothyroidism, and an unclear psychotic disorder who was admitted to an inpatient psychiatric unit on a 72 h hold after a suicide attempt by hanging. Jane grew up poor in the rural midwestern United States. She had numerous past psychiatric hospitalizations, but her primary diagnosis was unclear. Historically her psychosis and catatonia were most often attributed to bipolar disorder, however she also held diagnoses of major depressive disorder (MDD) with psychotic features and schizophrenia. On initial interview patient was fearful with significant psychomotor slowing, internal preoccupation and emotional lability. Ligature marks were evident around her neck. She described an intricate delusion involving local authorities and her family to end her life. She was reacting to internal stimuli and endorsing auditory hallucinations. Motives for her actions were unclear, she remembered the attempt but had minimal memory of the events prior and following. She was found wandering the streets in a stupor before being taken to the emergency department (ED). In the ED her urine drug screen was negative, and no alcohol was detected in her system. As interview progressed patient quickly became mute.

Most of history was obtained from chart review and family. Records showed patient’s first hospitalization occurred 10 years ago after Jane developed delirium from a urinary tract infection. She was agitated in the ED and required physical restraints. Following being restrained she quickly developed catatonia. She was admitted to psychiatry, diagnosed with bipolar disorder, and started on lithium. She spent the next decade in and out of psychiatric hospitals. She had numerous episodes of psychosis and catatonia. Countless treatment regimens were trialed, but patient progressively worsened. During one hospitalization where she was believed to have schizophrenia such high doses of antipsychotics were trialed that she developed rabbit syndrome. Family reported patient got in a fight with her daughter today which triggered her. Family reported history of irritability, sleep disturbances, episodic psychosis, hallucinations, and progressive delusions. Patient was noted to have no psychiatric history prior to her first marriage. Her ex-husband sexually abused her and was a member of the local authorities. Records also showed they abused crack-cocaine together though Jane never presented to the hospital for this and was in sustained remission for over three decades. Family stated Jane typically improved from hospitalization, but recently her illness seemed refractory to treatment.

## Differential diagnosis

1. Unspecified Psychosis
  (A) Bipolar Disorder, Unspecified Episode with Psychotic Features.
  (B) MDD w/ Psychotic Features.
  (C) Schizophrenia.
2. Recurrent Akinetic Catatonia
3. R/O PTSD
4. Rabbit Syndrome

It was clear on admission patient had an episodic psychotic disorder with associated sleep disturbances and catatonia. She had no prodrome, intact abstraction and periods of relative normalcy between episodes which suggested against schizophrenia. Patient did present with sleep disturbances, psychomotor slowing, and suicidal ideation (SI) but she explicitly denied depression endorsing anxiety and terror instead. Family did not endorse recent dysphoria or anhedonia. She did not meet the criteria for MDD, but as she had a longstanding history of episodes of reduced sleep, racing thoughts, distractibility, and psychomotor agitation bipolar disorder was considered the most likely diagnosis. This was also supported by a poor response to numerous serotonin reuptake inhibitors in the past as well as the presence of catatonia which is most commonly associated with bipolar disorder [6]. Her SI, psychomotor retardation and fatigue did suggest she may be in a mixed episode. Initially, timelines for symptomatology were not available to determine clearly if her symptoms were of sufficient duration to qualify for hypomania or mania. PTSD was considered likely, but the full extent of patient’s trauma history was not evident on admission. A rhythmic chewing motion was noted around her lips with no tongue fasciculations, confirming a prior diagnosis of rabbit syndrome.

## Treatment/managment

Jane was placed on suicide precautions and started on lithium and lurasidone for presumptive bipolar depression. She was also started on prazosin due to concern she may be having nightmares from PTSD. The patient’s local prescription drug monitoring system was checked which noted 3 months of prescribed clonazepam 0.5 mg twice daily (BID). She was clearly catatonic, so she was restarted on clonazepam initially but then transitioned to lorazepam due to lack of response. Her response to lorazepam was also poor. It improved her psychomotor slowing enough to allow her to eat and drink, but her mutism, dissociation and internal preoccupation seemed unchanged if not worsened. Clinician-Administered Dissociative States Scale (CADSS) was attempted at this time, but it could not be completed due to her mutism. An attempt was made to taper her lorazepam due to worsening derealization, but she decompensated into a fully vegetative state with waxy flexibility, stupor, and complete mutism. Lorazepam was restarted but while ramping up the dose she developed hypotension and even with discontinuation of prazosin and addition of fludrocortisone her blood pressure could not tolerate more than 2 mg of lorazepam three times daily (TID). CADSS scale still could not be completed, and her catatonia remained. Jane had expressed during past hospitalizations that she did not want ECT, and family asked we respect her wishes and to stabilize her with psychopharmacological interventions only.

A decision was made to trial oral zolpidem due to several case reports suggesting efficacy as well as research suggesting it may have a preferable hemodynamic profile compared to some benzodiazepines [8, 13]. After only one dose Jane was spontaneously engaging with treatment team unmasking her lingering psychosis and eventually her PTSD. Lorazepam was stopped and zolpidem was continued while Jane was engaged in daily psychotherapy. Over the next several days Jane divulged to us that she was bound and raped in her first marriage and developed flashbacks, nightmares, hypervigilance, and panic attacks from her trauma. Being restrained in the ED prior to her first psychiatric hospitalization caused her PTSD to flare up as it resembled being bound when she was abused. As her ex-husband was involved in law enforcement patient never sought legal charges and slowly grew to distrust the law. Security in the hospital and officers serving mental inquest warrants often triggered her PTSD. It was believed that Jane’s psychosis and delusions all stemmed from this trauma network. Prior to admission Jane’s daughter told Jane that she should have died when she was raped by their father. This triggered her and she kept hearing a voice saying that a “bloodbath” is coming. She felt an attempt on her life was inevitable and attempted suicide to try and take control of her own fate.

Zolpidem was eventually ramped up to 10 mg TID which Jane tolerated easily. After a few days her catatonia appeared fully broken. Her psychosis and delusions slowly improved. She still expressed anxiety and paranoia but denied depression and mood symptoms, as she had the entirety of admission. CADSS was administered prior to discharge, and she scored a 17/92, suggesting minimal dissociative symptomatology. By discharge she had good insight into the possibility of her delusions being false, her hallucinations had remitted, and she felt safe to return home and work with her daughter to resolve their differences. She was discharged on zolpidem 5 mg TID, lithium 600 mg at bedtime and lurasidone 40 mg which were tapered off over a month.

Six months post discharge Jane is still doing well. She has made good progress in therapy. She has come to realize that living in the home that she suffered her past trauma has made it difficult to heal and move on. She is working with her daughter to sell their home and downsize into an assistive living facility. She is tapered off all medicines, including zolpidem and benzodiazepines. She is free of catatonia and has had no resurgence of her psychosis to date.

## Discussion

Catatonia is one of the few disorders in psychiatry that is a medical emergency. It can rapidly lead to death and diagnosis and treatment during early stages is imperative. To effectively treat catatonia the underlying cause of it should be elicited. It is our belief that Jane’s main diagnosis was missed for her entire life. Her history clearly suggested that she had PTSD but her trauma was never investigated. All of Jane’s symptoms can be explained solely from PTSD-SP, which can easily mimic numerous conditions. Jane experienced reduced sleep, racing thoughts, distractibility, and psychomotor agitation but these were thought to be due to sympathetic hyperarousal and anxiety. Per family and old records, there was no episode of manic or hypomanic symptoms of sufficient duration to suggest patient genuinely met the criteria for bipolar disorder as her longest episode without sleep lasted less than 48 h. Her persistent fatigue during her sleep deprivation, which was noted by patient, providers and family was also a strong sign that she was not truly bipolar. Patient may have seemed depressed on admission, but the emotional numbing, affective constriction, detachment and derealization of PTSD often appears like dysphoria. Jane persistently denied dysphoria even while feeling comfortable enough to say that she was anxious and divulge in detail her trauma history. She definitely did struggle with depression in the past, but this admission was not felt to be related to MDD. She was psychotic, but as her history of developing rabbit syndrome from high dose antipsychotic therapy suggested her psychosis was heavily misunderstood.

Our case’s rapid improvement from zolpidem after failing benzodiazepine therapy suggests that special considerations should be given to managing catatonia in patients with PTSD-SP. It is possible that z drugs such as zolpidem may be preferable to benzodiazepines for managing catatonia in this populace, either due to greater tolerability or better long-term outcomes. If PTSD-SP patients respond better to z drugs than benzodiazepines for catatonia, z drugs should be considered as an alternate first-line treatment. Zolpidem may also be effective for catatonia in non-psychotic manifestations of PTSD, but this is yet to be tested. This topic warrants further research and may provide insight into new directions for future management of both catatonia and PTSD-SP.

## Data Availability

I declare that the authors will make available all data and materials requested for auditing and review of the above manuscript and research conducted. This includes but is not limited to signed inform consent documents, formal evaluation checklists and medical records as needed. Dr. Bonomo (NWBono01@louisville.edu) should be contacted for individuals requesting this data.
